# Association of Dental College Faculties

**Published:** 1885-10

**Authors:** L. P. Bethel


### THE DENTAL REGISTER.

VOL. XXXIX.] OCTOBER, 1885. [No. 10.

### PROCEEDINGS.

### Association of Dental College Faculties.

REPORTED BY L. P. BETHEL, D.D.S.

The Second Annual Meeting of the National Association of Dental Faculties was held at the Sherman House, Chicago, Ill., on Friday and Saturday, July 31st and August 1st. The colleges represented were as follows:

Baltimore—R. B. Winder, M. W. Foster.
Pennsylvania College of Dental Surgery—C. N. Peirce.
Philadelphia College—F. H. Guilford, Jas. Trueman.
University of Penn., Dental Department—E. T. Darby.
New York College of Dental Surgery—F. Abbott.
Boston College of Dental Surgery—J. A. Follet.
Ohio Dental College—H. A Smith.
University of Michigan—J. A. Watling, J. Taft.
Vanderbilt University—W. H. Morgan.
Iowa University—A. O. Hunt, L. C. Ingersoll.
Kansas City Dental College—J. D. Patterson.
Chicago Dental College—T. W. Brophy, A. W. Harlan.

MORNING SESSION—FIRST DAY.

The meeting was called to order at 11 o'clock A.M. by the President, Prof. C. N. Peirce, of Philadelphia.

The minutes of the previous meeting were read and approved. Roll-call was next in order, after which the Secretary read letters from the University of Maryland, acknowledging the receipt of an invitation to unite with the Association. The Faculty sanctioned the work of the Association, yet deemed it inexpedient to join, as they believed their instruction was superior to other colleges, etc.

By motion of Dr. Trueman, the letter was referred to the Executive Committee for consideration.

The next letter was from the St. Louis Dental College Faculty, Drs. Fuller and Eames stating that they would subscribe to any rule or regulation the Association might adopt.

By motion of Dr. Winder, the Executive Committee was instructed to furnish an order of business to facilitate the work.

Dr. Abbott offered the report of the Committee regarding the order of business, which was as follows:

Reading of minutes.

Roll-call.

Reception of reports of committees.

Unfinished business.

New business.

Reception of new members.

Reports of officers.

Miscellaneous business.

Report adopted.

Dr. Abbott then presented the following thoughts that should be brought before the Association and acted upon:

Preliminary examinations.

Foreigners entering our colleges.

Admission to the Senior class.

Question of fees.

Final examination.

Post-graduates.

On motion of Dr. Abbott, a committee of three was appointed to take the subjects into consideration and arrange them.

President Peirce appointed Drs. Abbott, Winder, and Trueman.

It was then moved by J. Taft that when they adjourned they would do so to meet at 3 P.M. to hear reports of committees. Carried. Adjourned.

AFTERNOON SESSION—FRIDAY.

The meeting was called to order at 3 P.M.

The minutes of the morning session were read and approved.

The Secretary read a letter from the Illinois Dental College, of Chicago, stating that they had just received a charter, but had organized no faculty as yet, and wished to be informed as to the course of study they should adopt. By motion the communication was laid on the table.

A partial report of the committee was presented, and by motion the report was taken up and acted upon by sections.

The subject of "Preliminary Examinations" was brought up, and Dr. Taft moved the adoption of the report. This was seconded, and remarks made by different members.

*Dr. Taft* thought that the object of preliminary examinations was not to ascertain whether the student was or could become qualified to make a dentist; but, rather, has he the attainments that will allow him to proceed with the work before him?

The man who has been trained in study and mental culture is better qualified to grasp the subjects brought to his attention; and the man who has the best trained mind is the one who is the best qualified to grapple with the scientific part of the profession. He who has not mind training or education has to learn many things by mere intuition, or mechanically. Those men who are ignorant should not enter the profession, or we will eventually not have a profession, that better requirements would have secured.

*Dr. Ingersoll* (Ia.): I heartily endorse Dr. Taft's statements. If we do not start men higher up, how can we expect to graduate them higher up? What we want is men with disciplined minds who are qualified after special preparation to enter the dental profession.

*Dr. Patterson* favored the idea of the faculties making out a list of questions each year for the different colleges.

*Dr. Foster* did not favor this suggestion.

*Dr. Taft* moved that the action taken last year be referred to a special committee, who should take the matter in charge and suggest the particular branches and the feasibility of getting up questions for the colleges, and report at the morning session.

Drs. Taft, Foster, and Ingersoll were appointed as a committee.

The regular order of business was suspended, and the applications for membership taken up.

*Dr. Taft* proposed the Dental College of Vanderbilt University, of Tennessee.

*Dr. Morgan*, of Vanderbilt University, said that owing to circumstances now existing they could not enter the Association before next year, but they would then be ready to abide by its rulings, and hoped the Association would admit them at that time.

Referred to a special committee for consideration, on which they reported as follows:

"*Resolved*, That in accordance with the statements of Prof. W. H. Morgan, of Vanderbilt University, Dental Department, this Association extends to him an invitation to participate in its deliberations, and will most heartily welcome the school which he represents, as a member of this Association whenever in its judgment it shall apply for such admission."

The Executive Committee would here state that the application for admission as a member of this Association has been made by the Vanderbilt University, Dental Department, but under the circumstances the Committee has concluded to not act upon it until our next meeting, in which decision Prof. Morgan fully concurs.

FRANK ABBOTT,
J. TAFT,
JAS. TRUEMAN.

Report accepted and adopted.

Regular business was now taken up, and the following resolution was read and adopted:

"*Resolved*, That applicants for admission to our Senior classes from foreign countries shall be required to furnish properly attested evidence of study, attendance on lectures, etc., the same as is required of the Junior students, and pass the intermediate examinations.

Adjourned.

SATURDAY MORNING—SECOND DAY.

Meeting called to order at 9:40.

Minutes of previous session read and approved.

It was recommended that the fees of all colleges, as far as practicable, be made uniform. Recommendation adopted.

It was then recommended that a committee of three be elected to settle questions that arise between sessions. On motion it was adopted.

The following committees were then recommended: Executive, Curriculum, and Text Books. Report adopted.

The President appointed the following:

Executive Committee—Abbott, Truman, and Taft.

Committee on Curriculum—Taft, Brophy, and Ingersoll.

Committee on Text Books—Winder, Hunt, and Guilford.

One hour recess was allowed for committees to prepare reports.

Reassembled at 11:30.

The Committee on Curriculum reported on preliminary examinations.

"*Resolved*, That a preliminary examination be required for entrance to our dental colleges; such requirements shall include a good English education. In case of any applicant failing to pass a satisfactory preliminary examination, the other colleges of this Association may be informed of the fact.

"*Resolved*, That a candidate for matriculation who presents a diploma from a reputable literary institution, or other evidence of literary qualification, shall be admitted without further examination."

Report adopted as follows:

"*Resolved*, That as prefatory to the preliminary examinations we recommend the following questions:

"1. Write your name in full.

"2. Give date of birth. Day, month, year.

"3. Where were you born? Town, county, state.

"4. Where do you now reside? Town, county, state.

"5. What educational advantages have you had? Name the schools you have attended, and the time spent in each.

"6. What branches have you studied, and to what extent have you pursued them?

"7. In what occupation have you been engaged other than that of student, and how long thus employed?

"8. When did you commence studying dentistry? Month, year.

"9. How many months of actual medical and dental study have you had to date?

"10. Have you attended a full course at any medical or dental school? If so, where and when?

"11. With what preceptor or preceptors have you studied? Give name and present residence.

"Sign name in full.

"Write an English composition of at least two hundred words upon a subject of the examiners' selection.

"Further examination left to the judgment of the different faculties. The scope of examinations proposed is as follows: (1) English Grammar; (2) Arithmetic; (3) Geography; (4) Modern History; (5) Government topics. A failure to pass examinations may be communicated to other colleges."

*Dr. Taft* moved that a committee of three be appointed to draw up a schedule of the different branches and questions upon each and forward to the different colleges. Carried.

Committee consisting of Drs. Taft, Ingersoll, and Harlan was appointed.

In regard to admission of students into the Senior class, the following resolutions were adopted:

"*Resolved,* That the colleges of this Association will receive into the Senior class only such Juniors as hold certificates of having passed a satisfactory examination in the studies of Junior year; this certificate to be a pledge to any college to which they may apply that a previous term has been properly spent in the institution whence they came.

"*Resolved,* That the certificate shall read as follows:

"This is to certify that............has attended one full course at the...........College of Dental Surgery. He was absent from lectures ........weeks. He was absent from practical work......... weeks. He was examined at the close of the session and found ............to enter the Senior class.

"*Resolved,* That each student, upon completing one regular course in any college represented in this Association, shall be furnished with the above certificate, without presentation of which he shall not be accepted by any of our colleges for admission to the Senior class."

*Dr. Wilson* then made the following motion:

That a committee of three be appointed to take into consideration the advisability of having a uniform practice in the furnishing of equipments for clinical work. Carried.

The committee consisted of Drs. Watling, Wilson, and Darby.

*Dr. Taft* moved that the motion to meet biennially be reconsidered. Carried.

It was then moved that the meeting be held one year hence. Carried.

Committee on Text Books then made a report which was discussed at some length, after which Dr. Taft moved that a committee composed of one member of each college faculty in the Association be appointed to take in charge the whole matter of text books and submit the report at the next regular meeting; also that they have discretionary power, so long as they do not bring any pecuniary responsibility upon the Association. Carried.

The Committee on Text Books was selected as follows: Baltimore, R. B. Winder; New York, F. Abbot; Michigan, J. Taft; Boston, J. A. Follet; Penn. Unniversity, J. Trueman; Iowa, L. C. Ingersoll; Missouri, W. H. Eames; California, S. W. Dennis; Kansas City, J. D. Patterson; Philadelphia, F. H. Guilford; Ohio, H. A. Smith; Penn. College, C. N. Peirce; Chicago, A. W. Harlan.

Treasurer's report was read and referred to Auditing Committee, who reported favorable. Report adopted.

Moved by Dr. Abbott that the assessment be made three (3) dollars from each faculty. Carried.

Election of officers followed. C. N. Peirce, President; R. B. Winder, Vice-President; H. A. Smith, Secretary; H. W. Harlan, Treasurer. Executive Committee consisting of Abbott, Trueman, and Taft was appointed for the coming year.

Reports of the proceedings were ordered printed.

No further business being presented, the meeting adjourned to meet one year hence.

